# Supplementary material for: Clinical features and outcome of Streptococcus agalactiae bone and joint infections over a 6-year period in a French university hospital
Source: PLoS One. 2021 Mar 12;16(3):e0248231. doi: 10.1371/journal.pone.0248231 (PMC7954318; doi:10.1371/journal.pone.0248231)
Supplement: Attachment — Submitted filename: Response to Reviewers_PlosOne.docx [file pone.0248231.s002.docx]

**Response to Editor and Reviewers**

**Plos One**

**PONE-D-20-36123**

**Editor**

**1. Please ensure that your manuscript meets PLOS ONE's style requirements, including those for file naming. The PLOS ONE style templates can be found at**

**https://journals.plos.org/plosone/s/file?id=wjVg/PLOSOne_formatting_sample_main_body.pdf and**

**https://journals.plos.org/plosone/s/file?id=ba62/PLOSOne_formatting_sample_title_authors_affiliations.pdf**

*Authors’ response: We have checked these points.*

**2. Thank you for including your ethics statement:  "Approval from the Institutional review board of our university hospital was obtained (IRB N°15/06.07). All procedures performed in the study were in accordance with the ethical standards and with the 1964 Helsinki Declaration and its later amendments or comparable ethical standards. Informed consents have been obtained. No identification data are disclosed.".**

**Please amend your current ethics statement to include the full name of the ethics committee/institutional review board(s) that approved your specific study. Once you have amended this/these statement(s) in the Methods section of the manuscript, please add the same text to the “Ethics Statement” field of the submission form (via “Edit Submission”).**

*Authors’ response: As requested, the full name of the IRB has been added and the ethic statement moved to the materials and methods section.*

**3. Please provide additional details regarding participant consent. In the ethics statement in the Methods and online submission information, please ensure that you have specified what type you obtained (for instance, written or verbal, and if verbal, how it was documented and witnessed). If your study included minors, state whether you obtained consent from parents or guardians.**

**For additional information about PLOS ONE ethical requirements for human subjects research, please refer to http://journals.plos.org/plosone/s/submission-guidelines#loc-human-subjects-research.**

*Authors’ response: As requested, we have specified the type of consent we obtained*

**4. Please include the date(s) on which you accessed the databases or records to obtain the data used in your study.**

*Authors’ response: As requested, dates on which we accessed the records to obtain the data were added in the materiel and methods section.*

**5. Please report any exclusion criteria used to select patients in your study.**

*Authors’ response: As requested, exclusion criteria has been added.*

Page 3, line 71 : Those with non-bone and joint infection were excluded.

**6. Thank you for stating the following financial disclosure:**

**"The funders had no role in study design, data collection and analysis, decision to publish, or preparation of the manuscript"**

**At this time, please address the following queries :**

a. **Please clarify the sources of funding (financial or material support) for your study. List the grants or organizations that supported your study, including funding received from your institution.**
b. **State what role the funders took in the study. If the funders had no role in your study, please state: “The funders had no role in study design, data collection and analysis, decision to publish, or preparation of the manuscript.”**
c. **If any authors received a salary from any of your funders, please state which authors and which funders.**
d. **If you did not receive any funding for this study, please state: “The authors received no specific funding for this work.”**

**Please include your amended statements within your cover letter; we will change the online submission form on your behalf**.

*Authors’ response: As requested, we have clarified the financial disclosure.*

7**. Your ethics statement should only appear in the Methods section of your manuscript. If your ethics statement is written in any section besides the Methods, please move it to the Methods section and delete it from any other section. Please ensure that your ethics statement is included in your manuscript, as the ethics statement entered into the online submission form will not be published alongside your manuscript.**

*Authors’ response: As requested this change has been done.*

**Reviewer #1:**

**This manuscript is a retrospectively identified case series of 26 patients (62% with polymicrobial infection) with Streptococcus agalactiae bone and joint infections (BJIs), describing their clinical features and treatment outcomes from a single center.**

**2. The manuscript lacks of any novel information regarding epidemiology, clinical features, or treatment outcomes of bone and joint infections caused by S. agalactiae.**

*Authors’ response: The literature on bone and joint infections specifically due to GBS is scarce. To the best of our knowledge, the largest series to date is the one from Kerneis et al (Ref 11 in our article) published in 2017 including 163 patients. In this paper there are only patients’ description and data on resistances of the isolated strains of S. agalactiae. In the article from Seng et al. (Ref 10 in our article) presenting clinical features and outcomes of bone and joint infection with Streptococcus involvement there is no specific data on the treatment used and clinical outcomes of the 37 GBS bone and joint infections included. Conversely to these two works, our manuscript displays specific information on antibiotics treatment used and clinical outcomes in mono (Table 2) and polymicrobial (Table 3) GBS BJI patients.*

**3. The conclusions in the abstract are not supported by data presented.**

*Authors’ response: We have made the following changes in the conclusion of the abstract (see also comment 4 and 5)*

Diabetes mellitus was the most common comorbidity. We observed an heterogenous management and a high rate of relapse.

**4. "S. agalactiae BJIs mainly affected diabetic patients" - In a case series with no comparison group, this statement is unwarranted. A more appropriate statement would be "Diabetes mellitus was the most common comorbidity among these patients."**

*Authors’ response: We have made the change suggested by the reviewer.*

**5. "Treatment failure was frequent, probably due to the complexity of BJIs requiring a multidisciplinary management." There is no statistical analysis presented to support the statement that complexity of BJIs requiring multidisciplinary management was associated with treatment failure.**

*Authors’ response: As suggested by the reviewer we have changed this sentence by the following : “*We observed an heterogenous management and a high rate of relapse.”

**6. "The most common risk factors were tobacco use (10, 38%) followed by alcohol abuse (5, 19%)." Risk factors for what?**

*Authors’ response: We agree with the reviewer that this sentence is not very clear and is furthermore not relevant for the topic . We have decided to remove this sentence from the text and the term “risk factor” from Table 1.*

**Reviewer #2**:

We had pleasure to read the article which is rich of informations that help to better understand the BJIs in the context of a french teaching Hospital. The methodology was rigorously performed and results are clearly shown and verifiable. In order to increase more the value of the article, we had some suggestions that doesn’t affect substantively the study but just the shape.

**1- The first time the acronym PTI appear in the article, it doesn’t follow by the signification. So it will be better to explain so early the explanation of the abbreviation.**

*Authors’ response: As requested by the reviewer, we have added the meaning of PJI in the abstract.*

**2- The rate of deaths appear in the article to be 2/25 or the actual patients included in the study is 26. The explanation of why you used 25 rather than 26 appear at line 173 with the one patient lost of follow up.
The authors should look how to early inform this in the results of the study.**

*Authors’ response: The total of 25 is only used for clinical outcomes and this is where we chose to explain that one patient was lost to follow-up. We think it might be confusing to explain the lost to follow-up earlier in the results section as the first parts of the results are displayed on 26 patients.*
